# Acute tea tree oil intoxication in a pet cockatiel (*Nymphicus hollandicus*): a case report

**DOI:** 10.1186/s12917-020-2255-4

**Published:** 2020-01-31

**Authors:** Alessandro Vetere, Mara Bertocchi, Igor Pelizzone, Emanuele Moggia, Chiara Travaglino, Mariangela Della Grotta, Silvia Casali, Sebastiano Gerosa, Laura Strada, Katia Filia, Jacopo Casalini, Enrico Parmigiani, Francesco Di Ianni

**Affiliations:** 1 Clinica Veterinaria Modena Sud, Modena (MO), Italy; 2 Department of Veterinary Science, University of Parma, Parma (PR), Italy; 3 Ambulatorio Veterinario Levante, Chiavari (GE), Italy; 4 Ospedale Veterinario San Francesco, Milano (MI), Italy; 5 Clinica Veterinaria Dott. Mario Crippa, Barzago (LC), Italy

**Keywords:** Intoxication, Tea tree oil, Cockatiel, Pet bird

## Abstract

**Background:**

Phytotherapy is becoming a more and more common practice, not only for personal care but also for pet care. Nevertheless, we often have to deal with substances on which, in most cases, very little literature is available, even more so if the species of interest are the exotic ones. In particular, the essential oil from the Melaleuca leaves, because of its antinflammatory and antibacterial properties, is widely used and very little is known about its potential toxicity on pet birds. The present paper describes the first case of Tea tree oil intoxication in a pet bird.

**Case presentation:**

A one-year-old, 80 g male cockatiel (*Nymphicus hollandicus*) was presented for clinical examination due to a serious despondency episode after the application of 3 drops of tea tree oil (*Melaleuca alternifoglia*) directly on the cutis of its right wing. The subject was urgently hospitalized and blood tests were performed.Serum biochemical values showed severe liver damage and slight renal involvement, complete blood count (CBC) parameters indicated a moderate neutrophilia a moderate neutropenia. Warm subcutaneous fluids and vitamin (VIT) B12 were administered, and after 8 h of fluid therapy the clinical condition of the patient improved. The subject was discharged after 48 h of hospitalization, in stable conditions.

**Conclusions:**

Toxicosis are relatively common in bird pets and a number of cases are reported in literature, concerning heavy metals intoxications and toxic plants ingestion. However, in literature there are no described cases regarding Melaleuca oil intoxication in pet birds, but it has been reported in humans (mainly by ingestion) as well as in dogs, cats and rats. We hope that this first case report can be an initial aid in the knowledge of this potential toxicosis and therefore in the clinical veterinary practice of pet birds.

## Background

Toxicoses are relatively common in bird pets. Indeed, a number of cases are reported in literature concerning both heavy metal intoxications and toxic plants ingestion [1–3]. Birds are smart and curious animals, therefore the accidental risk of toxic substances ingestion is relatively high. The body response to toxic agents is variable. It depends basically on size and weight of the subject, on the body nutrition status and on the amount of inhaled or systemically absorbed toxins [3]. In pet birds, deadly Polytetrafluoroethilene (PTFE,Teflon™) fume intoxications are not uncommon. In general, clinical signs depend on various factors, for example, nature of the toxin, route of exposure, amount of toxin adsorbed and if the poisoning is acute or chronic [4]. *Melaleuca* is a genus of more than 200 species of endemic plants in Australia and Malaysia [5]. *Melaleuca alternifolia*, otherwise called “Tea tree” is an Australian species from the northern coast with a high content of terpinen-4-ol (more than 30% of gross weight) and low content of cineole (lower than 15%) [6]. The essential oil from the Melaleuca leaves, because of its antinflammatory and antibacterial properties, is widely used in the traditional medicine among the native Australian population to treat infections of the urinary tract, fingernails, skin, and acne [7]. Due to its components, such as terpinen-4-ol, α-terpineol, linalool, α-pinene, β-pinene, β-myrcene and 1,8-cineole, tea tree essential oil has demonstrated an high degree of antimicrobial effect against a wide range of bacteria (*Staphylococcus aureus*, *Staphylococcus epidermidis*, *Streptococcus spp*, VRE-vancomycin resistant enterococci, *Acinetobacter baumanni*, *Escherichia coli*, *Klebisella pulmonae*), fungi (*Candida spp*, *Malassezia spp*) and protozoa (*Trichomonas vaginalis*) [6–9]. Tea tree has also been used in a spray form to control ticks (*Ixodes ricinus*) and poultry red mites (*Dermanyssus gallinae*) in poultry houses [9]. Terpenes are a class of organic compounds produced by a variety of plants [10] and animals, expecially some insects [11]. The use of synthetic and natural terpenes in medicine is widely documented. Epidemiological studies suggest that dietary monoterpenes may be helpful in the prevention and therapy of cancers [12–15], fungal [16], bacterial [17], and parasitic diseases [18, 19]. Despite of the facts, literature about the use of this substances as therapeutics in veterinary medicine is still lacking [20]. There are some papers about the toxicity of Melaleuca alternifolia and its extract [21, 22], but only few reports about the toxicity of other terpenes and their metabolites.

## Case presentation

**Fig. 1:**
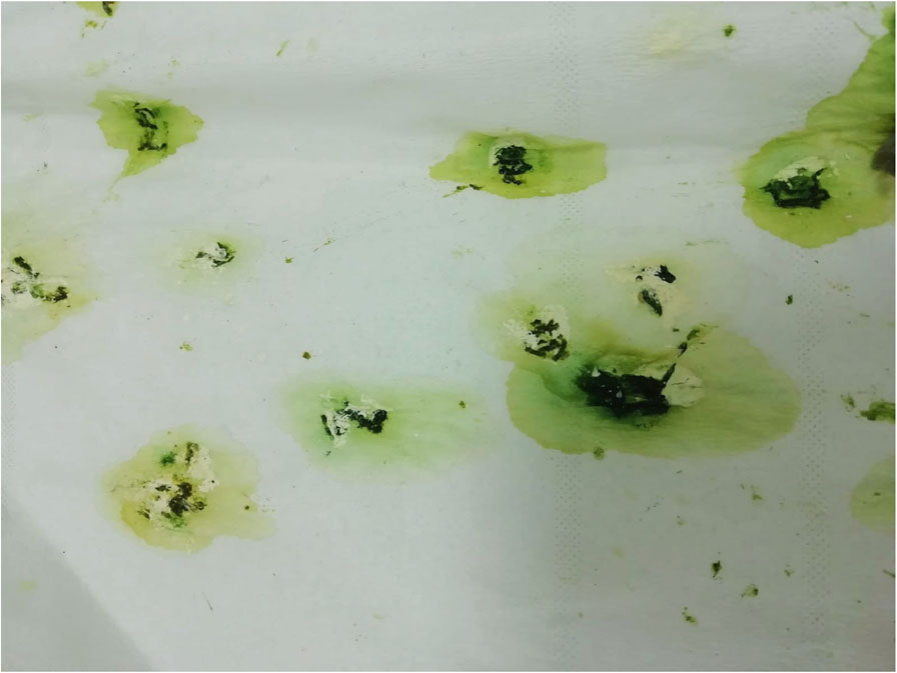
Fecal appearance after 8 h hospitalization. The brilliant green colors of the biliverdin in the urates are a result of liver damage

A one-year-old, 80 g male cockatiel (*Nymphicus hollandicus*) was presented for clinical examination due to a serious depression episode lasted for about 4 h, which occurred after 3 drops (0,15 ml) of 100% pure tea tree oil (*Melaleuca alternifoglia*) were applied directly on the cutis of its right wing (humeral region) and part of the thorax. The parrot was properly managed: it was kept in a cage of about 80x40x50 cm, equipped with perches and water dispensers, and its diet was based on seeds, fruits and pellets. The owner reported that, before entering a comatose state, the parrot suffered convulsions and vomit that lasted about 15 min. The symptoms appeared 30 min after the essential oil was applied. Upon physical examination, the subject was comatose, it did not react to stimuli and was bradypneic (36 breaths/min) [23]. The affected area of the skin was hyperaemic. The subject was urgently hospitalized and placed under oxygen in a warm room at 28 °C (100% sat, 4 tl/min). A 2 projectional radiography (latero-lateral, LL, and dorsal-ventral, DV) was performed and revealed no sign of change in coelomic organs. A 0.8 ml venous sampling from the jugular vein was performed and then sent to the laboratory in order to evaluate the biochemical parameters, which showed severe liver damage and slight renal involvement, compared to references values reported in Table 1 [IDEXX Laboratories Italia S.r.l.; 23]. Moreover, the CBC parameters highlighted a slight heterophilia (84%) compared to reference values (40–70%) [24]. Warm subcutaneous fluids (Ringer solution) (50 ml/kg) [25] and VIT B12 (Dobetin 500 μg/ml injectable Via Amelia 70, 00181, Roma, Italia) (0.5 mg/kg IM [26]) were administered. After 8 h, the breathing normalized (65/min) [23] and the subject recovered its normal position. The state of consciousness was no longer comatose but only reduced and the patient started to eliminate faeces with a slightly diminished consistency and with bright green urates (Fig.1). After about 4 h, the bird started to spontaneously feed and was given hepatoprotector (Birdetox©, DRN srl, Via Bellisario, Cremona,Italia.) at the dosage of 1 ml/kg live weight orally and fluid therapy SC (2 ml ringer + Vit b12) once a day (SID). The subject was discharged after 48 h of hospitalization, in stable condition. The therapy prescribed was only the aforesaid hepatoprotector at 1 ml/kg for at least 1-month therapy. A follow-up visit after a week was arranged, in order to repeat the relevant blood biochemical tests. The subject was re-examined after the two-week therapy. It was alert and its main organic functions worked properly. The faeces were normal in consistency and colour. A new blood sample was taken from the jugular vein, in order to re-examine biochemical and CBC parameters. Aspartate Aminotransferase (AST), Creatinkinasis, Lactic Acid Dehydrogenase (LDH) compared to previous values, as shown in Table 1. The CBC was within the normal range [27].

**Table 1:** Comparison between biochemical values and CBC (Complete Blood Count) parameters of the patient (first blood sample, taken upon patient admission) and the reference values. The table shows as well the comparison between patient’s biochemical values at admission to that of post treatment (after the two-week therapy)

	Patient’s values at admission	Patient’s post treatment values	Reference Values
AST (Aspartate aminotransferase) (U/l)	742	328	100–350 ^a^
Bile acids (μmol/l)	30.8	39.8	Colorimetric assay: 44–108 ^b^
Total protein (g/l)	23	26	24–48 ^b^
Albumin (g/l)	8	10	7.8–17.5 ^b^
Creatine kinase (U/l)	4806	450	30–245 ^a^
LDH (Lactic Acid Dehydrogenase) (U/l)	955	200	125–140 ^a^
Phosphorous (mmol/l)	1.6	1.2	1–1.5 ^a^
Calcium (mmol/l)	1.9	2.1	2.1–3.2 ^a^
Potassium (mmol/l)	2.6	3.0	2.5 – 4.5 ^a^

^a^ IDEXX Laboratories Italia S.r.l., ^b^ [23]

*Hb* Hemoglobin, *MCV* Mean corpuscular volume, *MCH* Mean corpuscular hemoglobin and *MCHC* Mean corpuscular hemoglobin concentration were not evaluated

## Discussion and conclusions

With exotic animals it is not uncommon to encounter to encounter diseases due to wrong management, for example due to an improper diet [28, 29]. Phytotherapy is undoubtedly becoming a more and more common practice, not only for personal care but also for pet care. In herbalist shops, products derived from plants are often sold as safe and free of side effects. For this reason, pet owners sometimes improvise an initial treatment using these products, or turn to the vet asking if it is advisable to use an herbal product to treat their animals. Consequently, we often have to deal with many substances for which, in most cases, very little literature is available, even more so if the species of interest are the exotic ones. Essential tea tree oil is one of these: most products found on the market are not registered for use on pets, while some others are specifically marketed as products for pet hygiene and care (above all for dogs and cats). With respect to its toxicity the *Melaleuca* essential oil is similar to some other essential oils containing terpenes, such as eucalyptus oil. Tea tree oil intoxication has been reported in humans (mainly by ingestion) as well as in dogs, cats and rats: the latter show signs of sense depression, weakness, obtundation, incoordination, ataxia and muscle tremors [21, 22, 30, 31]. Intoxication in cats entails an increase in the liver enzymes, thus highlighting the hepatotoxicity of *Melaleuca* oil. The increase in blood urea nitrogen (BUN) and dehydration, also in cats, does not exclude renal damage [22]. This could be due to mainly hepatic metabolism of terpenes and to the fact that they are mainly excreted through urines [31]. This case shows similar signs of intoxication, with severe liver damage and mild renal involvement, confirmed by the results of biochemical examinations. The toxicity of tea tree oil after oral intake is well documented in both experimental studies in rats (rats oral LD50: 1.9–2.6 ml/kg), and from cases of tea tree oil poisoning in humans [21, 32]. Toxicity following dermal application of high-doses of tea tree oil is reported in dogs and cats. For example, is documented the intoxication of three cats treated for flea with 120 ml of 100% pure TTO to their shaved skin [33]. The tree tea oil contains over 100 components and there is no information on the specific components responsible for the toxicity [21]. Although the rapid absorption through skin and digestive tract of tea tree oil is due to the highly lipophilic nature of the terpenes, no data are available about the absorption of each component when part of the mixture that constitutes the essential oil [21]. In our case is it possible that the toxic effects were due to both transdermal and gastrointestinal absorption, as the preening habits typical of parrots could have facilitated the ingestion of the essential oil. A definite diagnosis would be possible with the identification and quantification of terpenes in urates. Treatment in our case, included immediate supportive therapy through SC fluids and a proper source of heat. It was also worth checking the blood biochemical parameters after 2 weeks, to confirm recovery of organ functioning. In the literature numerous cases are reported in relation to pet bird toxicosis, due to heavy metals and toxic plants. In psittacines also Avocado (Persea spp.) intoxication has been reported [34, 35]. However, there are no described cases regarding Melaleuca oil intoxication in pet birds, while it has been reported in humans, dogs, cats and rats. The toxicology risk associated with herbal and natural remedies is not negligible. These types of products are uncontrolled, untested, unlicensed in-spite-of being very pharmacologically active. It is therefore important to inform veterinarians who can educate pet owners.

## Data Availability

All data generated or analyzed during this study are included in this published article [and its Additional file].

## Abbreviations

AST: Aspartate aminotransferase
BUN: Blood urea nitrogen
CaEDTA: Calcium disodium ethylenediamine tetraacetate
CBC: Complete blood count
Cu: Copper
DV: *Dorsal*-*Ventral*
Hb: Hemoglobin
HCT: Hematocrit
IM: Intramuscular
LDH: Lactic Acid Dehydrogenase
LL: Latero-lateral
MCH: Mean corpuscular hemoglobin
MCHC: Mean corpuscular hemoglobin concentration
MCV: Mean corpuscular volume
Pb: Lead
PTFE: Polytetrafluoroethilene
RBC: Red blood cells
SC: Subcutaneous
SID: Once a day
VIT: Vitamin
WBC: White blood cells
Zn: Zinc

## References

[1] Harrison JG, Harrison GJ, Harrison LR (1986). Toxicology. Clinical avian medicine and surgery.

[2] Murphy LA (2015). Environmental toxicology: considerations for exotic pets. J Exot Pet Med.

[3] Harrison JG, Lightfoot T (2006). Clinical avian medicine.

[4] Raidal SR, Jeanesh SM (2006). Acute poisoning of silver gulls (*Larus novaehollandiae*) following urea fertilizer spillage. Avian Pathol.

[5] Cowley KJ, Quinn FC, Barlow BA, Craven LA (1990). Contributions to a revision of Melaleuca (Myrtaceae). 7-10. Aust Syst Bot.

[6] Carson CF, Hammer KA, Riley TV (2006). Melaleuca alternifolia (tea tree oil) a review of antimicrobial and other medicinal properties. Clin Microbiol Rev.

[7] EMA/HMPC. Assessment report on Melaleuca alternifolia (Maiden and Betch) Cheel, M.linarifolia Smith , M.dissitiflora F.Mueller and /or other species of Melaleuca, Aetheroleum. Eur Med Agency. 2013. https://www.ema.europa.eu/en/documents/herbal-report/draft-assessment-report-melaleuca-alternifolia-maiden-betch-cheel-m-linariifolia-smith-m/other-species-melaleuca-aetheroleum_en.pdf. Accessed 10 July 2019.

[8] Nimbarte S, Kulkarni A (2013). Comparitive phytochemical analysis and resilience pattern exhibited by thyme and tea tree oil against selected poultry isolates. J Agric Vet Sci.

[9] Puvača N, Čabarkapa I, Petrović A, Bursić V, Prodanović R, Soleša D, Lević J (2019). Tea tree (Melaleuca alternifolia) and its essential oil: antimicrobial, antioxidant and acaricidal effects in poultry production. Worlds Poult Sci J.

[10] Langenheim JH (1994). Higher plant terpenoids: a phytocentric overview of their ecological roles. J Chem Ecol.

[11] Breitmaier E. Terpenes: Flavors, Fragrances, Pharmaca, Pheromones. Weinheim: Wiley; 2006. ISBN 978-3527317868

[12] Paduch R, Kandefer-Szerszeń M, Trytek M, Fiedurek J (2007). Terpenes: substances useful in human healthcare. Arch Immunol Ther Exp.

[13] Carvalho CC, Fonseca MM (2006). Carvone: why and how should one bother to produce this terpene. Food Chem.

[14] Kris-Etherton PM, Hecker KD, Bonanome A, Coval SM, Binkoski AE, Hilpert KF, Griel AE, Etherton TD (2002). Bioactive compounds in foods: their role in the prevention of cardiovascular disease and cancer. Am J Med.

[15] Gupta A, Myrdal PB (2004). Development of a perillyl alcohol topical cream formulation. Int J Pharm.

[16] Parveen M, Kamrul H, Junko T, Yoshinori M, Emiko K, Osamu K, Hitoshi I (2004). Response of Saccharomyces cerevisiae to a monoterpene: evaluation of antifungal potential by DNA microarray analysis. J Antimicrob Chemother.

[17] Trombetta D, Castelli F, Sarpietro MG, Venuti V, Cristani M, Daniele C, Saija A, Mazzanti G, Bisignano G (2005). Mechanisms of antibacterial action of three monoterpenes. Antimicrob Agents Chemother.

[18] Robledo S, Osorio E, Munoz D, Jaramillo LM, Restrepo A, Arango G, Velez I (2005). In vitro and in vivo cytotoxicities and antileishmanial activities of thymol and hemisynthetic derivatives. Antimicrob Agents Chemother.

[19] Uys AC, Malan SF, van Dyk S, van Zyl RL (2002). Antimalarial compounds from Parinari capensis. Bioorg Med Chem Lett.

[20] AbouLaila M, Sivakumar T, Yokoyama N, Igarashi I (2010). Inhibitory effect of terpene nerolidol on the growth of Babesia parasites. Parasitol Int.

[21] Hammer KA, Carson CF, Riley TV, Nielsen JB (2006). A review of the toxicity of Melaleuca alternifolia (tea tree) oil. Food Chem Toxicol.

[22] Carson CF, Riley TV (1995). Toxicity of the essential oil of Melaleuca alternifolia or tea tree oil. J Toxicol Clin Toxicol.

[23] Hawkins MG, Sanchez-Migallon Guzman D, Beaufrère H, Lennox AM, Carpenter JW, Carpenter JW, Marion CJ (2018). Birds. Exotic animal formulary.

[24] Campbell TW, Campbell TW (2015). Appendix B: Hematologic Values. Exotic Animal Hematology and Citology.

[25] Avanzi M, Crosta L, Peccati C, Selleri P (2008). Diagnosi e terapia delle malattie degli animali esotici. Coniglio, furetto, pappagalli, tartarughe.

[26] Samour J, Samour J (2000). Appendix 8, pharmaceutics commonly used in avian medicine. Avian Medicine.

[27] Speer BL, Speer BL (2016). Appendix 2, Normal clinical pathologic data. Hematology: laboratory reference ranges for selected species. Current Veterinary Therapy in Avian Medicine and Surgery.

[28] Legendre LFJ (2003). Oral disorders of exotic rodents. Vet Clin North Am Exot Anim Pract.

[29] Pelizzone I, Di Ianni F, Volta A, Gnudi G, Manfredi S, Bertocchi M, Parmigiani E (2017). Computed tomographic features of incisor pseudo-odontomas in prairie dogs (Cynomys ludovicianus). Vet Radiol Ultrasound.

[30] Nicholson SS (1995). Toxicity of insecticides and skin care products of botanical origin. Vet Dermatol.

[31] Villar D, Knight MJ, Hansen SR, Buck WB (1994). Toxicity of Melaleuca oil and related essential oils applied topically on dogs and cats. Vet Hum Toxicol.

[32] Russell M, Southwell I, Lowe R (1999). Toxicology of tea tree oil. Tea tree: the genus Melaleuca.

[33] Bischoff K, Guale F (1998). Australian tea tree (Melaleuca alternifolia) oil poisoning in three purebred cats. J Vet Diagn Investig.

[34] Craigmill AL, Eide RN, Shultz TA, Hedrick K (1984). Toxicity of avocado Persea americana (Guatemalan var) leaves: Reviev and preliminary report. Vet Hum Toxicol.

[35] Hargis AM, Stauber E, Casteel S, Eitner D (1989). Avocado (*Persea americana*) intoxication in caged birds. J Am Vet Med Assoc.

